# Chemistry of the Metals—Mercury

**Published:** 1854-07

**Authors:** Reginald N. Wright


AMERICAN JOURNAL

OF

DENTAL SCIENCE.

Vol. IV. NEW SERIES—JULY, 1854. No. 4.

### ORIGINAL COMMUNICATIONS.

### ARTICLE I.

*Chemistry of the Metals—Mercury.* By Professor REGINALD N. WRIGHT, A. M., M. D.

(Continued from page 346.)

WE present to our readers, a metal which has been known from remote antiquity. It is found both *native* and *mineralized;* in the former condition, however, (native mercury,) it occurs in but in few localities, and in comparatively minute quantities.* The principal ore of mercury, is that known commonly as *cinnabar*, a sulphuret of the metal, some very beautiful specimens of which have recently been found in California.

The lustre of mercury is eminently metallic, resembling polished silver, and this circumstance, added to the fact, that at ordinary temperatures it is fluid, has given origin to the

*It may not be amiss to remark, that in almost every mercury mine, occasional globules of the metal are found in a condition of almost absolute purity.

name, *hydrarguron*,* which literally translated from the Greek, means *silver-water.*

"The principal mines from which it is obtained are those of Idria in Carniola, and Almaden in Spain, where it is found both in the native state and combined with sulphur, as cinnabar, the latter being most abundant."—(Turner.) Considerable quantities have recently been found in California.

In procuring the metal from the sulphuret for ordinary purposes, we have but to apply heat, and add some substance which will retain the sulphur; we can use for this purpose either *iron* in minute division (as iron filings) or lime; the sulphur remaining in combination, while the mercury is volatilized and carefully condensed.

In Aikin's Chemical Dictionary, the processes of *Idria* and *Almaden*, are thus described: "At the mines of Deux Ponts and Idria, the ore being brought out of the mine, is sorted by hand with considerable accuracy, rejecting those parts which appear to be destitute of the metal. This is an expensive process, but has superseded the ancient method of separating the cinnabar by washing, on account of the prodigious loss of the metal by that operation. The sorted ore, being reduced to powder, is carefully mingled with 1-5th (more or less according to the proportion of cinnabar contained in the ore) of quicklime, which has fallen to powder by exposure to the air. This mixture is then put into iron retorts, capable of holding about sixty pounds weight, which, when thus charged, are fixed in a long furnace to the number of forty or fifty; a glass receiver being then attached to each retort, (but not luted,) a gentle fire is applied in order to drive out all the moisture; when this is effected, the juncture of the vessels is closely stopped with tempered clay, and a full read heat is applied for seven or eight hours, at the expiration of which time all the mercury will have been volatilized and condensed in the receivers. The common product varies from between six and ten ounces of metal, from one hundred pounds of ore."

* Hydrargyrum, is the term most commonly used.

"The method practiced at Almaden in Spain, differs considerably from the preceding, and is much more rude and inartificial. The pieces of pure cinnabar being first picked out from the ore, in order to be disposed of to the painters and manufacturers of sealing-wax, the rest is sorted into three parts. The first is the richest, and is in pieces of a moderate size; the second is in smaller pieces, and less abounding in metal; the third is the dust, and smallest fragments of the other two, which are kneaded up with clay, and formed into bricks, which are carefully dried in the sun. The furnace used for extraction of the mercury is an oblong mass of masonry, divided horizontally into an upper and lower compartment by an iron grate, and communicating near the top with a set of aludels. The charging of the furnaces commences by laying on the grate a stratum of flat rough stones, leaving intervals between each for the passage of the fire; upon this is laid a bed of ore of the second quality, then the ore of the first quality, afterwards another bed of the second kind, and at the top of all a layer of the third kind, made up into bricks. A few faggots are now thrown into the lower cavity of the furnace and lighted, which, in proportion as they are consumed, are succeeded by others, and thus a gentle fire is kept up for eight or twelve hours, according to the previous dryness of the ore. When the moisture is got rid of, (which is known by the cessation of the vapor,) the fire-place is again filled with the faggots, and, before these are consumed, the mass of ore will be sufficiently heated to continue the combustion, by means of the sulphur that it contains, without any additional fuel. During the next two days, (as the sulphur burns away,) the mercury, in the state of vapor, passes into the aludels, where it is condensed, and, at the end of this period, all the metal being extracted, the scoriæ are taken out of the furnace, and the aludels are emptied of their contents. Besides the mercury, a considerable quantity of a black matter like soot is found in the aludels, which is readily separated by spreading the whole about on an inclined table; the mercury runs to the lower end, where it is collected in a channel, while the impurities remain behind."

"The consumption of fuel, and cost of apparatus, is considerably less than in the German method, but it is probable that a portion of mercury still remains in the ore. A great loss is also sustained by throwing away the soot, after separating the running mercury on the tables, for not only many globules of mercury must escape notice, but also the calomel, cinnabar, &c. which it contains, are entirely wasted. This soot, previously to the separation of the mercury, consists, according to Proust, of

| | |
|---|---|
| Mercury, | 66 |
| Calomel, | 18 |
| Cinnabar, | 1 |
| Water and sulphurous acid, | 2 5 |
| Sulphate of ammonia, | 3 5 |
| Lamp black, | 5 |
| Selenite, | 1 |
| | 97 0 |
| Loss, | 3 |
| | 100 0 |

That mercury was well known to the ancients, is shown upon reliable testimony. We find in an English periodical, the following: "Pliny says, (xxxiii, 7,) that Callias, an Athenian, discovered the preparation of vermilion or cinnabar, B. C., 505. He also mentions the mines of Almaden, as producing in his time 10,000 Roman pounds annually, but this was not the amount which the mines could have produced, for the supply was purposely limited. LePlay, a French geologist, who visited Almaden in 1833, describes the mines as being richer than at any former period, furnishing annually nearly 2,244,000 pounds of mercury. About 700 men are employed under ground, and 200 in the operations connected with the extraction of the metal from the ore at the surface."

The method adopted for packing the metal after its reduction was peculiar, and much more imperfect than that in use at the present day, (wrought iron bottles being now almost universally used, containing each about 90 pounds.) According to Aikin,

"a fresh sound sheepskin, with the hair taken off, is laid over a wooden bowl, and from 50 to 75 pounds of mercury are poured into it; the ends of the skin are then gathered up and tied together with great care, thus forming a sort of bag in which the metal is enclosed; this bag is enclosed in a second skin, and the second in a third; lastly, these bags are put into very tight barrels, capable of holding from two to four of them, and in this state are brought to market." The following table, from Dumas, shows the present annual yield of the principal mercury mines of the world,* for quintals.†

| | | | |
|---|---|---|---|
| Almaden, | 25,000 | to | 32,000 |
| Idria, | 6,000 | " | 10,000 |
| Hungary, Transylvania, | 700 | " | 700 |
| Deux Ponts, | 400 | " | 500 |
| Palatinate, | 180 | " | 200 |
| Huancavelica, | 3,000 | " | 3,000 |
| Quintals, | 35,280 | " | 46,400 |

The equivalent number of mercury is variously stated, but may be put down at 200; its specific gravity is 13.5, which is increased to 14, when the metal solidifies. At common temperature, it is liquid, evaporates at 670°, solidifies with contraction at 40°, at which temperature it is malleable. The alloys of mercury and other metals, commonly known as *amalgams*, will next be presented, and since Professor Brande has, with his usual ability and accuracy, prepared an excellent paragraph upon the subject, we will lay it before our readers without comment or modification.

"Mercury combines with most of the other metals, and forms a class of compounds, generally called *amalgams*. Many of these are definite and crystallizable compounds, and may be separated by gentle pressure from the mercury, in which such definite compound is suspended or dissolved. They are gene-

* Mercury has been found in California, not mentioned in Dumas' list, because of its recent discovery.

† A quintal is nearly 108 avoirdupois pounds.

rally brittle or soft. One part of potassium with 70 of mercury, produce a hard brittle compound. If mercury be added to the liquid alloy of *potassium* and *sodium*, an instant solidification ensues, and heat enough to inflame the latter metals is evolved. Iron and mercury may be combined by triturating together clean *iron filings* and *zinc-amalgam*, and adding a solution of pure chloride of iron; by rubbing and heating this mixture, the iron and mercury form a bright amalgam.—(Arthur, Aikin.) Under common circumstances, iron resists the action of mercury so perfectly, that the latter metal is usually kept in iron bottles; and mercurial triangles and barometer cisterns are made of the same metal. The use of an amalgam of *zinc* has already been adverted to for the excitation of electrical machines.* Eight parts of mercury and one of zinc form a white brittle compound; five of mercury and two of zinc, form a crystallizable amalgam. Amalgam of tin is easily formed by triturating the metals together, or fusing them by a gentle heat; its density exceeds the mean of its components; it is largely used for silvering looking-glasses. This beautiful process is performed as follows: A single and perfect sheet of tin-foil, of proper thickness, and somewhat larger than the plate of glass, is spread upon a perfectly plane table of slate or stone; mercury is then poured upon it, and rubbed upon its surface with a hare's foot, or a ball of flannel or cotton, so as to form a clean and bright amalgam; upon this, excess of mercury is poured, until the metal has a tendency to run off; the plate of glass, previously made quite clean, is then brought horizontally towards the table, and its edge so adjusted, as by gradually and steadily sliding it forward, to displace some of the excess of mercury, and float the plate, as it were, over the amalgam, the dross on its surface being pushed onwards by the edge of the glass, so that the mercury appears beneath it, with a perfectly uniform, clean and brilliant reflecting surface; a number of square weights, of 10 or 12 pounds each, are now placed side by side upon the plate, so as entirely to cover

*** Refers to a former part of his work on chemistry.**

it, and press it down upon the amalgamated surface of the tin; in this way the excess of mercury is partly squeezed out, and the amalgam is made to adhere firmly to the glass. The mercury as it runs off is received into a channel on the side of the table, (which is slightly inclined to facilitate the drainage,) and in about 48 hours the weights are taken off, and the plate is carefully lifted from the table and set nearly upright, by which the adhering mercury gradually drains off, and the solid crystalline amalgam remains, perfectly and uniformly adhering to the glass. *Cadmium* and mercury unite with great ease, and the *amalgam* crystallizes in octoïdra, when composed of 100 mercury + 28 cadmium, (an atom of each metal,) it fuses at 167°.—(Stromeyer.) The amalgams of *cobalt* and *nickel* have not been examined. Amalgam of *copper*, may be made as follows:—viz. a hot solution of *sulphate of copper*, add a little hydrochloric acid and a few sticks of *zinc*, and boil the mixture for about a minute, by this means the copper will be precipitated in the metallic state and in a finely-divided spongy form; take out the *zinc* from off the liquor, wash the copper with hot water, and pour upon it a little dilute *nitrate of mercury*, which will instantly cover every particle of copper with a coating of mercury; then add mercury to the amount of two or three times the weight of the copper and a slight trituration will combine them so far that the completion of the process may be effected by heating the mixture for a few minutes in a crucible. (Aikin's Dictionary, Art. Mercury.) *Lead* and mercury readily combine in all proportions; 3 parts of mercury and 2 of *lead*, form a crystallizable compound. *Antimony* amalgamates with difficulty, and forms a granular compound. *Bismuth* and mercury readily unite; 2 parts of mercury poured into 1 of melted *bismuth* form a compound which slowly solidifies and crystallizes. When mercury is combined with a little bismuth, *lead* may be added without greatly interfering with the fluidity of the compound. Dr. Thomson states that Brecher was the first who observed the remarkable fluidity of a mixture of 3 parts of mercury, 1 of *lead* and 1 of *bismuth*, and that it may be squeezed through leather without decomposition; it is used for silvering the inside of glass balls, which are previously made perfectly clean and warm. When mercury is adulterated, it is with these metals, but the facility with which it then oxydizes and the imperfect fluidity of its small globules, renders the fraud easy of detection. The action of mercury on the other metals which have been described has not been examined, with the exception of that or *tellurium* and *arsenic.* With *tellurium* it forms a granular compound, with arsenic a gray amalgam of 5 parts of mercury and 1 of *arsenic;* the metals required to be stirred together for some hours over the fire. Dumas suggests the examination of the action of *arsenimelted hydrogen,* on the chlorides of mercury."

"*Amalgam of Ammonium, Metallization of Ammonia.*—It was first observed by Berzelius and Pontin, that when mercury is negatively electrized in a solution of ammonia, or of an ammoniacal salt, or when an amalgam of *potassium* and *mercury* is placed upon *moistened sal ammonia,* the metal increases in volume, and becomes of the consistency of butter, an appearance, which has sometimes been called the *metallization of ammonia.* If mercury, which has been amalgamated with about the fiftieth part of *potassium,* be made the *negative electrode* in a solution of *sal ammoniæ,* the effect is produced to its greatest extent; the mercury puffs up to 80 or 100 times its original bulk, and if, in this state, it be cooled to 32°, it crystallizes in cubes, the amalgam is lighter than water, but when left to itself, it gradually shrinks back again into mercury, evolving *ammonia* and hydrogen, not exceeding in weight, a *seventy-thousandth* of that of the amalgam, and yet conferring upon it the semi-fluid or solid state, and susceptibility of crystallization; according to Gay Lussare and Thenard, the volume of the *hydrogen* is 347, and of the *ammonia* 422, to 100 volume of mercury. These extraordinary phenomena, have suggested several hypothesis concerning the nature of ammonia and its components, and also of the metals, respecting which, the reader may consult Gay Lussare and Thenard, (Recherches Physico-Chimique, vol. i,) who, finding the amalgam resolvable into *mercury, ammonia* and *hydrogen,* regard it merely as a compound of these substances; and Berzelius (Lehrbuch, I,) who considers the appearances as resulting from the combination of a metal, which he terms *ammonium*, with the *mercury*; this view of the subject gave rise to what has been called the *ammonium theory*."

*Compounds of Mercury with Oxygen.*—Mercury combines with oxygen so as to form two oxyds known as *protoxyd and peroxyd*.

*Protoxyd of Mercury.*—Whenever one of the proto-compounds of mercury in solution, is acted upon by an alkaline solution, or when such a compound is rubbed with an alkaline solution, decomposition ensues, attended with the formation of the oxyd referred to. Under ordinary circumstances, when mercury is simply exposed to the action of the air, it undergoes no change.

Aikin has in his Dictionary the following: "Mercury is scarcely, if at all, changed by mere exposure to the air for any length of time, but if it be long agitated in any vessel it is slowly reduced to a black dust, soiling the fingers, which, however, on being heated returns again to the state of flowing mercury. Bœrhave was the first who performed this experiment, by fastening a bottle half full of mercury to a mill wheel, and thus giving it a very long continued and violent motion. The same has been done by fastening it to the wheel of a carriage during a long journey. Dr. Priestly found this black powder to be formed in a variety of circumstances by a long agitation of mercury in vials with water," &c.*

When we prepare the protoxyd by means of *protochloride of mercury* and *potassa* in solution, we must thoroughly wash the residuum, with cold water, after which it must be very carefully dried, and be kept during the latter process as much out of the light as possible, because, if incautiously exposed to the action of light, (especially the direct rays of the sun,) or even great heat, decomposition will happen, and metallic mercury will be deposited, and the peroxyd formed.

Speaking of the protoxyd of mercury, Brande has the follow-

*** When formed in this manner, it has been called "ethiops per se."**

ing: "A preparation often considered as nearly corresponding with this oxyd, is directed in the *London Pharmacopœa*, under the name of *hydrargini oxydum cinereum.* It is made by boiling calomel with lime-water; but it is not a pure protoxyd of mercury, and being uncertain in composition, is unfit for medical use. It is also supposed that the protoxyd of mercury is contained in the *pilula hydrargyri, or blue pill*, and in the *unguentum hydrargyri, or mercurial ointment.* But these preparations are rather to be regarded as containing *finely divided* mercury, and it is apparently obtained in a similar state when mercury is triturated with honey, mucilage, and other viscid bodies, and also with chalk, as in the *hydrargyrum cum creta* of the pharmacy. Indeed, it is astonishing to what an extent mercury may be subdivided into extremely minute globules, so as to lose its metallic appearance, even under a powerful magnifier; this is the case when solutions of mercury are precipitated by protochloride of tin; the metal then appears in the form of a gray powder, and remains in that condition while humid."

Some doubt still exists as to whether this substance is a suboxyd, protoxyd, or merely mercury, in a finely divided state; the weight of authority, however, is in favor of the existence of a true protoxyd, whose salts will yield a black precipitate with alkalies, and which will form proto-salts with acids. It has a decidedly metallic taste, cannot be dissolved in water, and has a very dark color.

The protoxyd of mercury may be represented by $Hg+O$.

*Peroxyd of Mercury.*—This oxyd is a red crystalline mass, having a strongly metallic taste, and almost insoluble in water.

One method of obtaining it consists in exposing the nitrate to a sustained heat. "In this process, twenty-five parts of mercury are dissolved in thirty-five parts of nitric acid. Nitric oxyd is abundantly disengaged, and on evaporation to dryness, a pure nitrate of mercury remains, which is carefully heated so long as fumes of nitrous acid are evolved. The resulting oxyd is in the form of an orange-red crystalline powder, forming the hydrargyri nitrico-oxydum of the pharmacopœa." (Brande, p. 931.)

There are several other methods of preparing this oxyd, but we will content ourselves with mentioning only the two following:

If mercury is heated to 600° and upwards, with free access of air, it will be formed abundantly, and quite pure; should any liquid mercury remain, we may get rid of it by evaporation at a higher heat.

The other method consists in precipitation from a solution of corrosive sublimate, by means of an alkali, and afterwards using heat to drive off water.

In order to detect adulteration, which is sometimes practiced, we may expose the peroxyd to high heat; if pure, there will be no residuum, but if mixed with other substances, as red lead, the latter will not be evaporated, and the fraud can be readily made certain.

This oxyd is represented by $Hg+2O$.

"Both the oxyds of mercury combine with the greater number of the acids, forming two distinct classes of salts, several of which are resolvable into salts, with excess of base, and salts with excess of acid, so that the history of the saline combinations of mercury is thus rendered somewhat complex. There is also a great tendency to the formation of double salts among the caloric mercurial compounds; in short, mercury is, of all the metals, that which produces the most numerous and complicated series of saline combinations.

[To be concluded in the next number.]

